# Photocatalytic Applications of Heterostructure Graphitic Carbon Nitride: Pollutant Degradation, Hydrogen Gas Production (water splitting), and CO_2_ Reduction

**DOI:** 10.1186/s11671-019-3070-3

**Published:** 2019-07-12

**Authors:** Williams Kweku Darkwah, Kivyiro Adinas Oswald

**Affiliations:** 1 Key Laboratory of Integrated Regulation and Resource Development on Shallow Lakes, Ministry of Education, College of Environment, Hohai University, 1 Xikang Road, Nanjing, 210098 People’s Republic of China; 2 Department of Science, Mkwawa University College of Education, University of Dar es Salaam, Dar es Salaam, Tanzania; 3 Department of Biochemistry, School of Biological Sciences, University of Cape Coast, Cape Coast, Ghana

**Keywords:** Photocatalysis, Heterostructure, Graphitic carbon nitride, Pollutant degradation, Hydrogen Gas Production, CO_2_ reduction

## Abstract

**Abstract:**

Fabrication of the heterojunction composites photocatalyst has attained much attention for solar energy conversion due to their high optimization of reduction-oxidation potential as a result of effective separation of photogenerated electrons-holes pairs. In this review, the background of photocatalysis, mechanism of photocatalysis, and the several researches on the heterostructure graphitic carbon nitride (g-C_3_N_4_) semiconductor are discussed. The advantages of the heterostructure g-C_3_N_4_ over their precursors are also discussed. The conclusion and future perspectives on this emerging research direction are given. This paper gives a useful knowledge on the heterostructure g-C_3_N_4_ and their photocatalytic mechanisms and applications.

**Impact Statements:**

The paper on g-C3N4 Nano-based photocatalysts is expected to enlighten scientists on precise management and evaluating the environment, which may merit prospect research into developing suitable mechanism for energy, wastewater treatment and environmental purification.

## Introduction

### Background of Photocatalytic Semiconductors

In 1972, Fujishima and Honda discovered the water photolysis on a TiO_2_ electrode [1] as the response to the steady increase of energy shortage and environmental pollution caused by industrialization and population growth in 1970 [2]. Their discovery was recognized as the landmark event that stimulated the investigation of photonic energy conversion by photocatalytic methods [2]. Due to population growth, high industrialization, and improvements in agricultural technologies, till the twenty-first century, energy shortage and environmental pollution are still challenges [3]. In recent decades, photocatalysis has become one of the most promising technologies owing to its potential applications in solar energy conversion to solve the worldwide energy shortage and environmental pollution alleviation [4]. Photocatalysis is the process that involves photocatalyst. “A photocatalyst is defined as a substance which is activated by adsorbing a photon and is capable of accelerating a reaction without being consumed” [5]. Photocatalysts are invariably semiconductors. Several semiconductors such as TiO_2_, ZnO, Fe_2_O_3,_ CdS, and ZnS are used as photocatalysts in environmental pollutants treatment and solar fuel production such as methane (CH_4_), hydrogen (H_2_), formic acid (HCOOH), formaldehyde (CH_2_O), and methanol (C_2_H_5_OH) [6]. Due to its photocatalytic and hydrophilic high reactivity, reduced toxicity, chemical stability, and lower costs [7], TiO_2_ has been mostly studied as having the high ability to break down organic pollutants and even achieve complete mineralization [8]. Due to its large band energy, TiO_2_ can only absorb solar energy in the UV regions which only constitutes 4% of the total solar energy irradiated [9, 10]

For efficient performance, a photocatalyst semiconductor requires a suitable band gap for harvesting light [11], facile separation and transportation of charge carriers (electron and holes) [12], and proper valence band (VB) and conduction band (CB) edge potential for redox reaction being thermodynamically feasible [13]. Several semiconductor modifications such as surface modification, metal doping, and heterojunctions formations have been taken to give the best photocatalytic activity of different photocatalyst semiconductors [14–16]. Also, the plasmon-enhanced sensitization was found to be effective in improving the photocatalytic activity efficiency of some photocatalyst [17, 18]. This is caused by the oscillation of electrons in the metal nanoparticle as a result of the induced electric field after solar irradiation, a term referred to as the localized surface plasmon resonance effects (LSPRs) [19]. In counteracting on the demerits of most inorganic photocatalyst such as visible light utilization, there has been a great increase of researches on the photocatalytic graphitic carbon nitride (g-C_3_N_4_) in recent decades due to its special structure and properties, such as its good chemical and thermal stability under ambient conditions, low cost and non-toxicity, and facile synthesis [20, 21]. Although some single g-C_3_N_4_ semiconductor photocatalysts demonstrated high photocatalytic efficiency on visible light illumination [22] compared to other photocatalysts like TiO_2_ [23], they suffer from high charger carrier (electron–hole pair) recombination which greatly reduce their photocatalytic efficiency [24] The construction of heterostructured photocatalyst systems comprising multicomponent or multiphase is one of most effective strategies to balance the harsh terms, owing to the tenable band structures and efficient electron–hole separation and transportation [25], which endow them with suitable properties superior to those of their individual components [26]. Several heterostructured semiconductor modifications have been studied over the three decades.

This paper, however, centers on the ability and efficacy of the prospective applications of construction of heterostructured carbon nitride to enhance the visible light-responsive photocatalytic performance of the candidate for energy, wastewater, and environmental treatment in order to project future implementations to elucidate environmental problems and related.

## Carbon Nitride

Presently, g-C3N4 is studied as a new-generation photocatalyst to recover the photocatalytic activity of traditional photocatalysts like TiO_2_, ZnO, and WO3. Graphitic carbon nitride (g-C3N4) is assumed to have a tri-s-triazine nucleus with a 2D structure of nitrogen heteroatom substituted graphite framework which include p-conjugated graphitic planes and sp^2^ hybridization of carbon and nitrogen atoms [27]. Bulky carbon nitride can be synthesized through thermal condensation of nitrogen-rich (without a direct C-C bound) precursors such as cyanamide, dicyandiamide, thiourea, urea, and melamine. Also, it can be synthesised through polymerization of nitrogen-rich and oxygen-free precursors (comprising the pre-bonded C-N core structure) by physical vapour deposition, chemical vapour deposition, solvothermal method, and solid-state reactions. Having the band gap of 2.7 eV and the conduction and valence band position at − 1.4eV and 1.3 eV, respectively, versus NHE (normal hydrogen electrode), g-C3N4 have shown great ability to carry photocatalytic activity in the visible light irradiation without the addition of any noble-metal co-catalyst [28]. Apart from visible light utilization, bulky carbon nitride is hampered by high-charge carrier recombination which reduces its photocatalytic activity. Different researchers have studied on the modification of g-C_3_N_4_ to counteract the challenge of charge carrier recombination and band engineering. Several modifications have been studied over decades including structural modification, doping, modification with carbonaceous and plasmonic material, and heterojunction composite formation.

### Structural Modification

Changing the morphology of the synthesized photocatalysts plays a significant effect in its photocatalytic activity [29]. Optical, electronic, mechanical, magnetic, and chemical properties of carbon nitride materials are highly dependants on the change of size, composition, dimension, and shape. Hard and soft templating methods, template-free methods, and exfoliation strategies are among the methods used to modify the structure of the synthesized carbon nitride photocatalysts [30]. Templating modifies the physical properties of carbon nitride semiconductor materials by varying morphology and introducing porosity. Template-free method creates vacancies in carbon nitride photocatalysts resulting to introduction of additional energy levels or acting as reactive sites, and thus profoundly changing the overall photocatalytic activity. Exfoliation modifies the bulky carbon nitride into nano-sheet carbon nitrides which increase the surface area for active sites, hence increasing its photocatalytic activity. Also, carbon nitride can be modified into nano-rods and nanotubes which all have effects on the photocatalytic activity of the synthesised photocatalyst.

### Doping

One and the most popular modification of a single semiconductor is the metal/non-metal doping [31] and surface modification forming metal/semiconductor-heterostructured photocatalysts [32]. Different researchers have studied doping g-C_3_N_4_ with different metals or non-metals for band gap engineering and overcoming the challenge of charge carrier (electrons-holes pair) recombination [33]. Yan et al. [34] reported the study on the impact of doped metal (Na, K, Ca, and Mg) on g-C_3_N_4_ for the photocatalytic degradation of enrofloxacin (ENR), tetracycline (TCN), and sulfamethoxazole (SMX) as representatives of common antibiotics under visible light irradiation. In their study, it was observed that in all the degradation of three representative antibiotics the degradation activity followed the same sequence of g-CN-K>g-CN-Na>g-CN-Mg>g-CN-Ca>g-CN. This was attributed by the decreased band gap of doped g-C_3_N_4_ from 2.57 to 2.29–2.46 eV as a result of a red shift caused by the doped metal resulting to an extended visible light response and high-charge carrier separation of the as-prepared photocatalytic semiconductor, hence increasing the production of ·OH reactive species [34]. In the study done by Xu et.al , it was also evident that doping Fe on the surface-alkalinized g-C_3_N_4_ reduced the recombination of photogenerated charge carriers (electron and holes) and the band energy of which lead to high photocatalytic activity of the doped g-C_3_N_4_ on the degradation of tetracycline under visible light (*λ* ≥ 420) irradiation [35]. Jiang et.al [36] synthesized the nitrogen (N) self-doped g-C_3_N_4_ nanosheets with tunable band structures for enhanced photocatalytic tetracycline degradation in the visible light irradiation. It was evidently proved that doping nitrogen (N) on g-C_3_N_4_ nanosheets increased the semiconductor photocatalytic activity as a result of reduced charge recombination as proved by the photoluminescence (PL) emission spectra study [36]. Ling et al. [37] reported the study of the synergistic effect of non-metal (sulphur) doping on the photocatalytic property of g-C_3_N_4_ using the first-principle calculations. The obtained results indicated narrowing of the band gap and increased visible light response on S-doped g-C_3_N_4_ photocatalyst [37]. The effect of metal or non-metal doping on g-C_3_N_4_ was also revealed in studies done by Guo et al. who used potassium (K) and iron (Fe) [38], Fan et al. who used manganese (Mn) [39], Xie et al., Zhu et al., and Wu et al. who used cobalt (Co) [40–42]. Shu et al. using sodium (Na) synthesized doped mesoporous g-C_3_N_4_ nanosheets for photocatalytic hydrogen production of which the results showed that the doped nanosheets exhibited lower recombination of photogenerated charge carrier (electron–hole pairs) than bulk g-C_3_N_4_, hence resulting to excellent visible light photocatalytic hydrogen evolution efficiency up to about 13 times that of bulk g-C_3_N_4_ [43]. All these prove that doping g-C3N4 with metal ion or non-metal has a significant improvement on the photocatalytic efficiency in the visible light irradiation.

### Modification with Other Carbonaceous Materials

Carbonaceous materials have a wide range of physical and chemical properties derived from the spatial organization of carbon atoms and their chemical covalent bonds [44]. Carbonaceous materials such as carbon nanotubes (CNTs), multiwalled carbon nanotubes (MWCNTs), carbon dots (CDs), graphene, and reduced graphene oxide have been widely incorporated in modifying different photocatalyst semiconductors in order to enhance their photocatalytic activity. Ma et al. [45] reported the synthesis of an artificial Z-scheme visible light photocatalytic system using the reduced grapheme oxide as an electron mediator. In their report, results showed that g-C_3_N_4_/RGO/Bi_2_MoO_6_ exhibited high photocatalytic activity (*k* = 0.055 min^−1^) over degradation of rhodamine B dye as one of the common pollutant [45]. Also, in 2017, Ma and coworkers reported the synthesis of Bi_2_MoO_6_/CNTs/g-C_3_N_4_ with enhanced debromination of 2, 4-dibromophenol under visible light. The composite resulted into higher photocatalytic activity (*k* = 0.0078min^−1^) which was 3.61 times of g-C_3_N_4_ (*k* = 0.00216 min^−1^) [46]. Xie et al. [47] reported the construction of carbon dot-modified MoO_3_/g-C_3_N_4_ Z-scheme photocatalyst with enhanced visible light photocatalytic activity for the degradation of tetracycline as one of the common antibiotic pollutant found in waste water. In their work, it was observed and proved that the composite exhibited higher photocatalytic activity where 88.4% of tetracycline was removed compared to only 5.3% removal of g-C_3_N_4_ [47].

### Heterostructure Graphitic Carbon Nitride Composite

The heterojunctions that are formed between the host semiconductors provide an internal electric field that facilitates separation of the electron–hole pairs and induces faster carrier migration [2]. It involve the combination of two semiconductors to form the heterojunction semiconductors.[48]. Several researches have proven that the heterojunction formation is the promising strategy to the improvement of the g-C3N4 photocatalytic activity.

According to the band gap and electronic energy level of the semiconductors, the heterojunction semiconductor can be primarily divided into three different cases: straddling alignment (type I), staggered alignment (type II), and Z-scheme system. The band gap, the electron affinity (lowest potential of CB), and the work function (highest potential of VB) of the combined semiconductors determine the dynamics of the electron and hole in the semiconductor heterojunctions [32]

- Type I heterojunction semiconductor

**Fig. 1:**
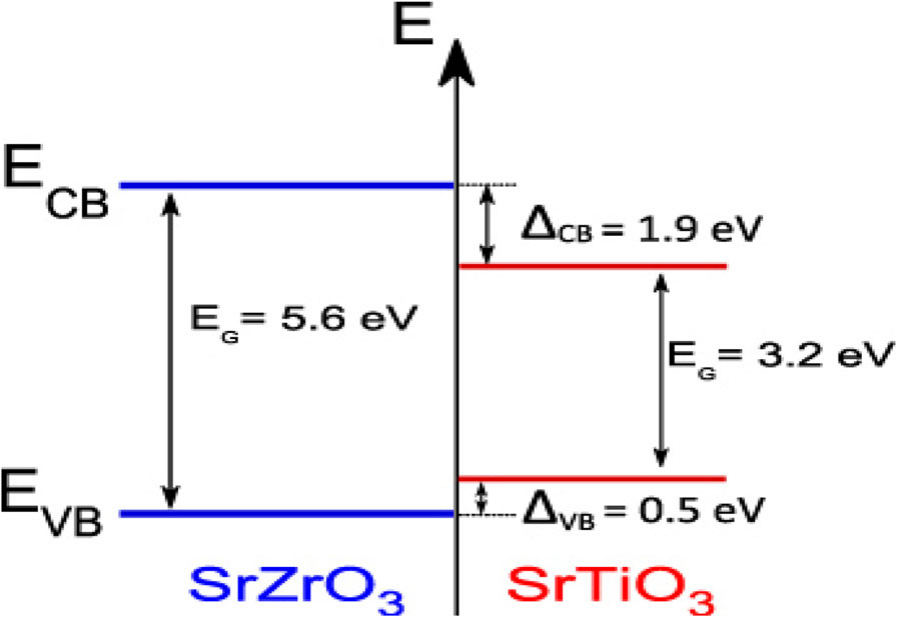
The systematic representation of the type I heterojunction semiconductor

In type-I heterojunction semiconductor, both VB and CB edges of semiconductor 2 are localized within the energy gap of semiconductor 1, forming straddling band alignment (Fig. 1). The VB and CB alignment play a significant role in the determination of the physical properties of the generated charges and the photocatalytic performance. This kind of heterojunction does not improve photocatalytic activity of the prepared photocatalyst because of the accumulation of both charge carriers on one semiconductor [49]. From Fig. 1, the photogenerated electrons (e^−^) are expected to move from the SrZrO_3_ conduction band (CB) to SrTiO_3_ conduction band (CB) due to reduction potential differences. Also, the photogenerated holes (h^+^) generated in the valence band (VB) of SrZrO3 will migrate to the valence band of SrTiO_3_ due to the difference in their oxidation potentials. Hence, both electrons and holes will accumulate in SrTiO_3_ semiconductor causing high recombination to take place.

(b) Type II heterojunction semiconductor

**Fig. 2:**
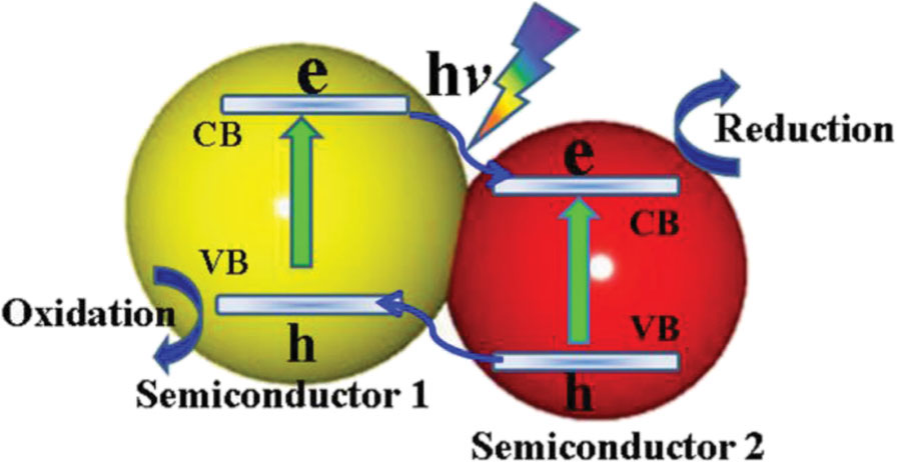
The systematic representation of the type II heterojunction semiconductor. Reproduced with permission [25]. Copyright 2015 WILEY-VCH Verlag GmbH & Co. KGaA, Weinheim

In type-II heterojunction semiconductor, both VB and CB of semiconductor 1 are higher than that of semiconductor 2 (Fig. 2). Electrons from semiconductor 1 migrate to semiconductor 2 while the holes move from semiconductor 2 to semiconductor 1. If both semiconductors have sufficient intimate contacts, an efficient charge separation will occur during light illumination. Consequently, charge recombination is decreased, and so charge carriers have a longer lifetime, which results in higher photocatalyst activity [32]. Type II heterojunction semiconductor suffer from steric hindrance of charge transfer. When electron in the CB of semiconductor 1 migrates to the CB of semiconductor 2, there is a repulsion force created between coming electrons and existing electrons. Same applies when holes from the VB of semiconductor 2 migrates to the VB of semiconductor 1. In the steric hindrance created, there can be a small amount reduction in the expected photocatalytic activity of the as-prepared type II heterojunction photocatalyst.

(c) Z-Scheme heterojunction semiconductor

**Fig. 3:**
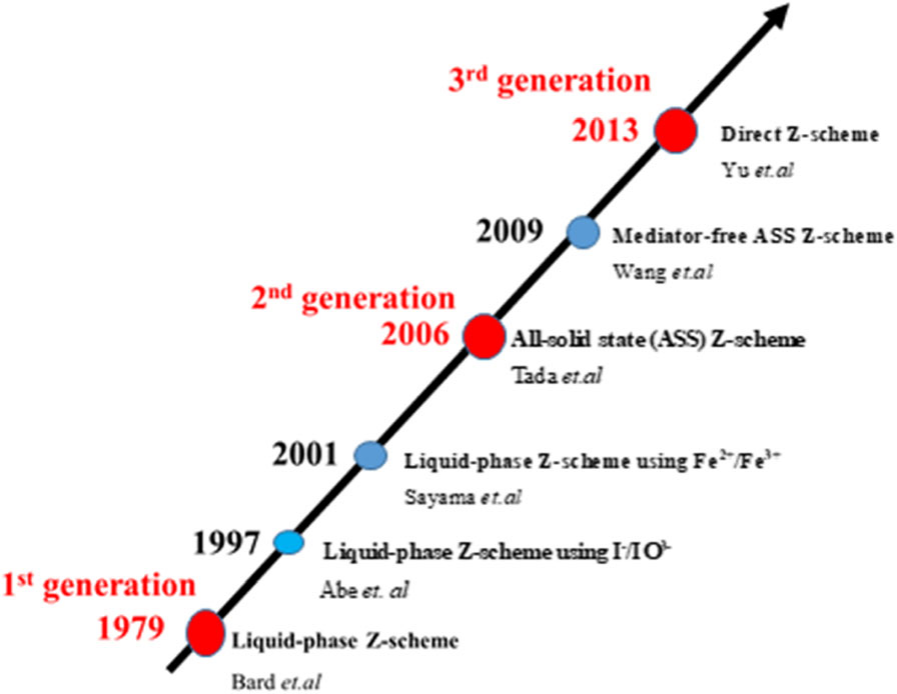
The roadmap of the evolution of z-scheme photocatalytic system. Reproduced with permission from [3] with slight modifications. Copyright 2017 WILEY-VCH Verlag GmbH & Co. KGaA, Weinheim

In the course of development and modifications of visible light-driven photocatalytic systems, Z-scheme was originally introduced by Bard in 1979 [32]. The Z-scheme heterojunction was developed to solve the steric hindrance exerted in type II heterojunction. Currently, there are three generations of the Z-scheme photocatalytic system (Fig. 3).

(i). First-generation Z-scheme heterojunction

**Fig. 4:**
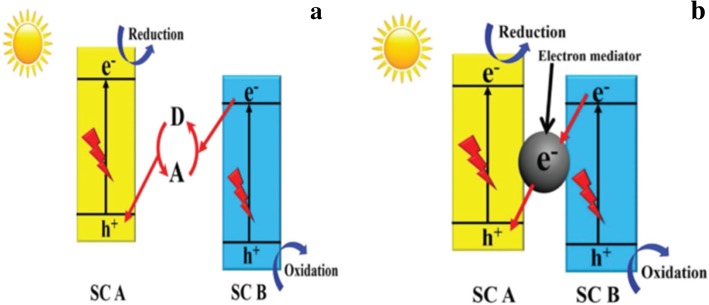
(**a**) A systematic representation of first Z-scheme generation where A and D are the electron acceptor and donor respectively. (**b**) A systematic representation of the second-generation Z-scheme (ASS). Reproduced with permission [3]. Copyright 2017 WILEY-VCH Verlag GmbH & Co. KGaA, Weinheim

It is also known as liquid-phase z-scheme photocatalytic system***.*** It is built by combining two different semiconductors with a shuttle redox mediator (viz. an electron acceptor/donor (A/D) pair) as seen in Fig. 4a. It was first proposed by Bard et.al in 1979. In 1997, Abe et al. synthesized the liquid-phase Z-scheme semiconductor using I^−^/IO^3−^, before Sayama et al. synthesised the liquid-phase Z-scheme using Fe^2+^/Fe^3+^ in 2001 [3]. Liquid-phase Z-scheme photocatalytic system can only be used for liquids. It also suffers from the backward reaction that is caused by the use of redox mediators such as I^−^/IO^3-^and Fe^2+^/Fe^3+^ [32].

(ii). Second-generation Z-scheme heterojunction semiconductor

It is also known as all-solid-state (ASS) Z-scheme system. In order to overcome the obvious problems identified in the first generation, Tada et al. in 2006 synthesised the all-solid-state CdS/Au/TiO_2_ Z-scheme [32]. An ASS Z-scheme photocatalytic system is composed of two different semiconductors and a noble-metal nanoparticle (NP) as the electron mediator as seen in Fig. 4b. The use of the noble metal solves the backward reaction that was happening in the first generation (liquid-phase Z-scheme). Noble metals are expensive and very rare to obtain causing their wide application to be limited. Also, noble metals have high ability to absorb light. This affects the light absorbance of photocatalytic semiconductors, and their photocatalytic activities are also affected. In solving the light absorbance problem, Wang et al. in 2009 synthesised the mediator-free ASS Z-scheme [3].

(iii). Third-generation Z-scheme heterojunction semiconductor

**Fig. 5:**
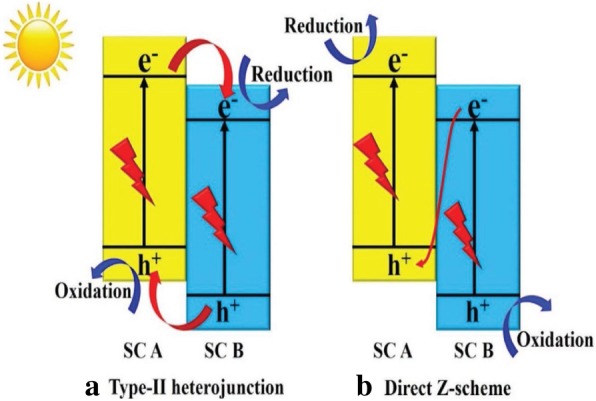
A comparison of the charge transfer between type II heterojunction (**a**) and Z-scheme heterojunction (**b**). Reproduced with permission [3]. Copyright 2017 WILEY-VCH Verlag GmbH & Co. KGaA, Weinheim

It is commonly known as direct Z-scheme semiconductor*.* A direct Z-scheme photocatalyst consists of only two semiconductors that have a direct contact at their interface [32]. All the advantaged features in the previous two generation are inherited in direct Z-scheme photocatalyst. Unlike a type II semiconductor, electrons in the CB of semiconductor B migrate to recombine with the holes generated in the VB of semiconductor A forming a Z-transfer as shown in Fig. 5**.** In order to facilitate the easy Z-transfer of charge carriers, the participating semiconductors must have a close band energy level with perfect CB and VB alignment [50]. Currently, this is the known and suitable heterojunction system with high charge carrier (electron and holes) separation efficiency.

## The Fundamental Mechanism of the Photocatalytic Semiconductor

**Fig. 6:**
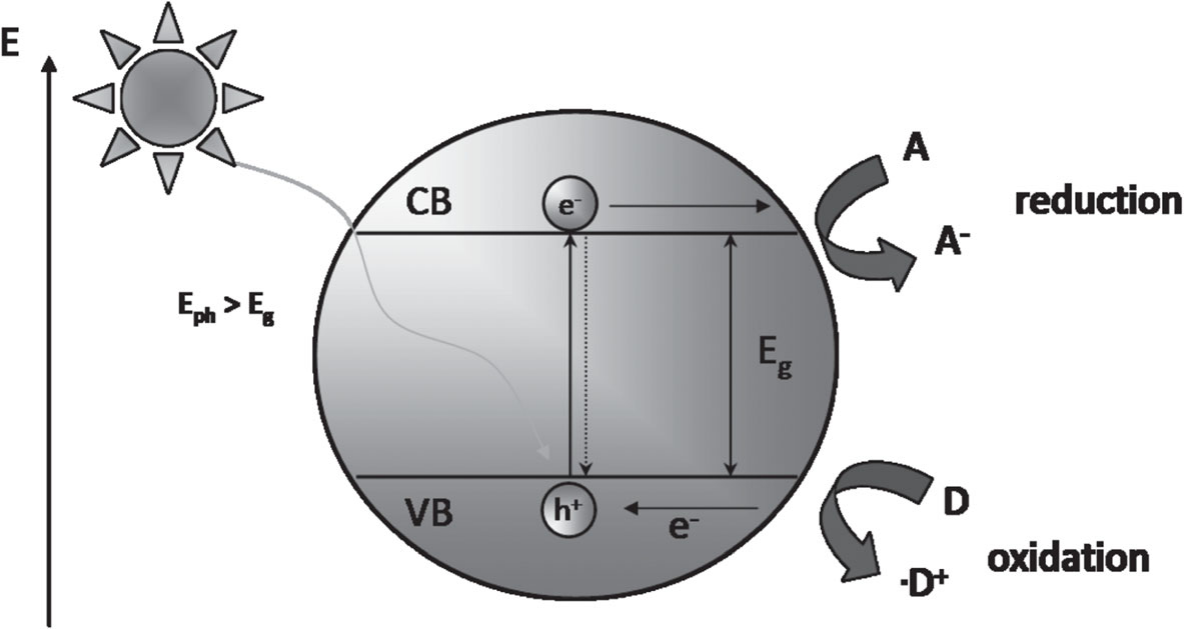
A systematic depiction of the general mechanism of photocatalytic semiconductor. Reproduced with permission [29]. Copyright 2013 WILEY-VCH Verlag GmbH & Co. KGaA, Weinheim

When the incident light photon with equal or large energy than the band gap energy strike the semiconductor, the electrons in the valence band (VB) are photoexcited and move to the conduction band (CB), leaving equal number of the holes in the valence band (VB) [21]. The photoexcited electron (e^−^) and holes (h^+^) in the CB and VB, respectively, moves to the surface of the semiconductor [51]. It is at the surface of the photocatalyst semiconductor where reduction and oxidation of the electron acceptor and electron donor, respectively, take place as seen in Fig. 6.

The photocatalytic mechanism is summarised by the following Eqs. 1, 2 and 3

1 $$ \mathrm{Semiconductor}+ hv\to {\mathrm{e}}^{\hbox{-}}\;\mathrm{CB}+{\mathrm{h}}^{+}\;\mathrm{VB} $$

2 $$ {\mathrm{e}}^{\hbox{-}}\;\mathrm{CB}+\mathrm{A}\to {\mathrm{A}}^{-} $$

3 $$ {\mathrm{h}}^{+}\;\mathrm{VB}+\mathrm{D}\to \cdot {\mathrm{D}}^{+} $$

The doping effect, surface modification, and heterojunction formation have the direct effect on the movement of the generated charge carriers (electron and holes) of the synthesized photocatalyst. When the electron mediator atom is introduced in the semiconductor, the movement of charge carrier depends on whether the mediator is an electron donor or acceptor. The dopant not only controls the charge recombination, but it also assists in band gap engineering of some wide band gape semiconductors. In heterojunction composite photocatalyst, the charge carrier transfer depends on the nature and properties of the participating semiconductors. In type II heterojunction semiconductor reduction and oxidation, reactions occur for semiconductor with a lower reduction potential and semiconductor with a lower oxidation potential, respectively. Due to electrostatic repulsion between electron–electron and hole–hole, the charge carrier transfer in type II heterojunction is restricted hence reducing photocatalytic activity of the synthesized photocatalyst. In the Z-scheme heterojunction, the movement of the charge carrier follows the Z-pattern where electrons remain on the semiconductor with the higher reduction potential while holes remain on semiconductor with the higher oxidation potential.

This paper place special emphasis on recently researched heterostructure graphitic carbon nitride (g-C_3_N_4_) looking at their characterizations and their applications in ambient conditions.

## Characterization Methods for Heterostructure g-C3N4

### Morphology

**Fig. 7:**
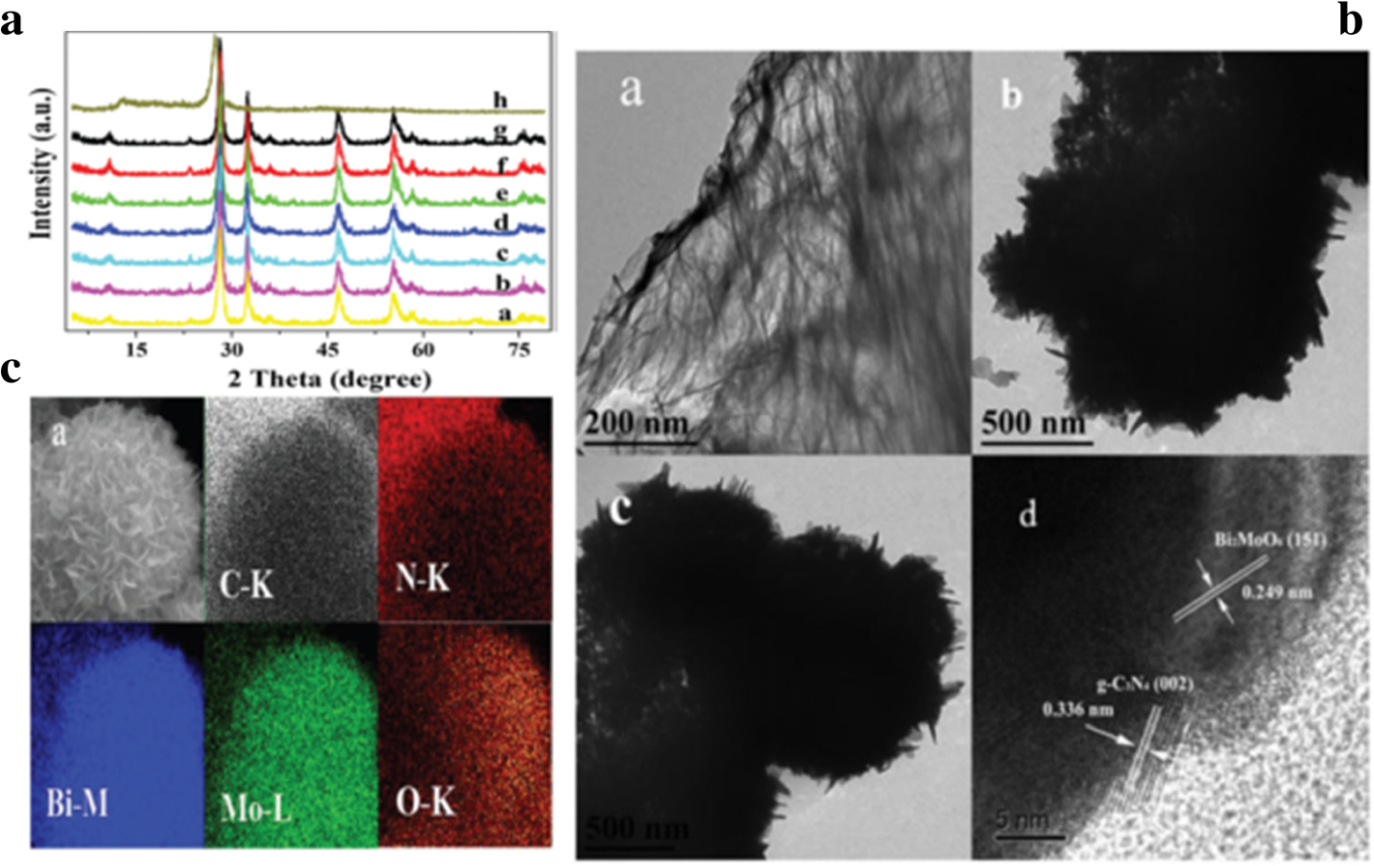
(**A**) XRD of (b–g) g-C_3_N_4_/Bi_2_MoO_6_ composites with different g-C_3_N_4_ content (a) Bi_2_MoO_6_ (h) g-C_3_N_4_ (**B**) TEM images of (a) g-C_3_N_4_ (b) Bi_2_MoO_6_ (c)g-C_3_N_4_/Bi_2_MoO_6_ composite (**C**) SEM image of (a) g-C_3_N_4_/Bi_2_MoO_6_ showing corresponding elemental (C, N, Bi, Mo, and O) mapping. Reproduced with permission [35]. Copyright 2014 Royal Society of Chemistry

The morphological structure of the synthesized photocatalyst plays a significant role in its photocatalytic activity [52]. SEM, TEM, and XRD are used to study the morphology of the as-prepared photocatalyst [53, 54]. XRD shows different peaks that confirms that the formed structures are in agreement with the standard cards [10]. SEM and TEM shows the morphology of the as-prepared photocatalyst [55]. Figure 7A shows the XRD spectra of g-C3N4 (h), Bi2MoO6 (a), and the g-C3N4/Bi2MoO6 composites (b–g). As seen, the peaks at 27.40° and 13.04° are corresponding to the (002) and (100) planes of g-C3N4 [56] while the peaks at 28.3°, 32.6°, 47.7°, and 55.4° are in agreement with (131), (002), (060), and (331) planes of Bi2MoO6, respectively [12] which shows the perfect formation of the g-C_3_N_4_/Bi_2_MoO_6_ composite. The existence of a uniform fringe interval (0.336 nm) in the TEM images (Fig. 7B) is in agreement with the (002) lattice plane of g-C_3_N_4_ while that of 0.249 nm is in agreement to the (151) lattice plane of Bi_2_MoO_6_. In the same shape (Fig. 7C) ascribed by the elemental mapping of C-K, N-K indicates the existence of g-C_3_N_4_ while the mapping of Bi-M, Mo-L, and O-K shows the existence of Bi_2_MoO_6_ in the as-prepared heterojunction. This proves that there were the perfect formation of the heterojunction between g-C_3_N_4_ and Bi_2_MoO_6_.

### X-Ray Photoelectron Spectroscopy (XPS) Characterization

The surface chemistry of the as-prepared composite has the greatest impact on its photocatalytic activity. X-ray photoelectron spectroscopy (XPS) characterization has been extensively used to determine the surface chemistry of materials [57] by studying the changes in the electronic density on the different surfaces of a photocatalyst through investigating the shift in the binding energies [58]. A shift in the binding energy of a specific element of the semiconductor is caused by the introduction of the foreign materials which affects the electron migration on its surface [25, 31]

**Fig. 8:**
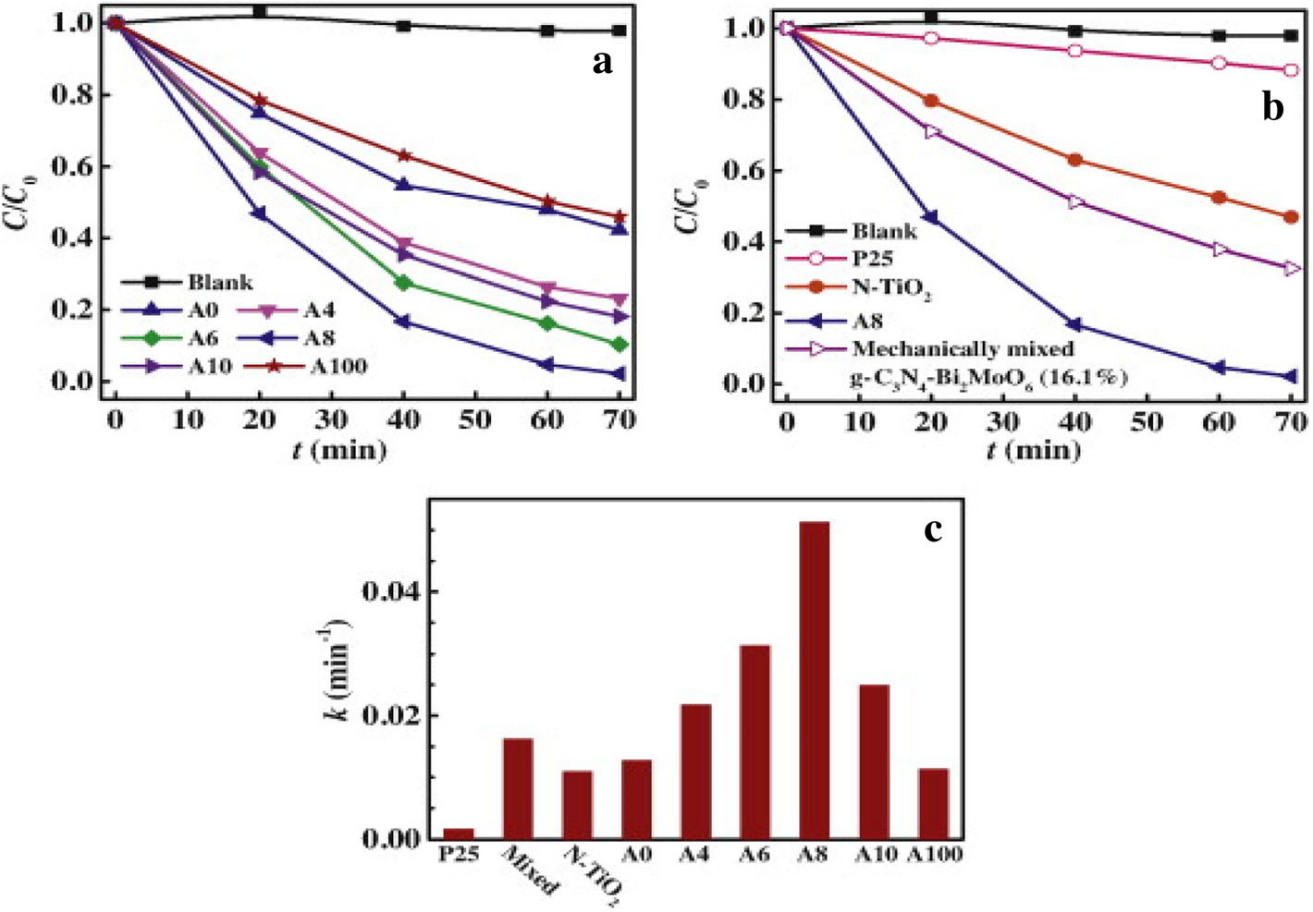
**a**, **b** RhB degradation over various photocatalysts and **c** corresponding rate constants (k). Reproduced with permission [23]. Copyright 2014 Elservier B.V

For instance, Longjun Song and coworkers confirmed the hydrothermal synthesis of novel g-C_3_N_4_/BiOCl heterostructure nanodiscs for efficient visible light photodegradation of rhodamine B using XPS characterization. In this study, all XPS spectra were calibrated using the C 1s signal at 284.8 eV [59]. The sp^2^-bonded carbon in N-containing aromatic rings (N–C=N) (Fig. 8b) were ascribed to the C 1s signals at 288.2 eV [60] while sp^2^-hybridized aromatic nitrogen bonded to carbon atoms (C=N–C) in triazine rings was attributed to 398.8 eV. This confirms the presence of sp^2^-bonded graphitic carbon nitride [60]. The existence of peaks at 159.4 and 164.5 eV is caused by Bi^3+^ in BiOCl while the peak at 530.2 eV is attributed to the Bi–O bonds in (BiO)^2+^ of the BiOCl. The weak peak at 404 eV is caused by the fact that g-C_3_N_4_ is coupled with BiOCl through the p-electrons of CN heterocycles. This confirms the coexistence of g-C_3_N_4_/BiOCl composite.

### Photocatalytic-Reduction Test

Not all the photogenerated electrons reaching the surface of the photocatalyst have the ability to carry the photocatalytic-reduction reaction. Only the photogenerated electrons with sufficient reduction potentials participate fully in the reduction reaction. Equations 4, 5, 5, 6, 7 and 8 summarize the standard redox potentials for various photocatalytic-reduction reactions.

4 $$ {\displaystyle \begin{array}{l}2{\mathrm{H}}^{+}+2{\mathrm{e}}^{\hbox{-}}\circledR {\mathrm{H}}_2,\\ {}{\mathrm{E}}_0=\hbox{-} 0.41\mathrm{V}\ \mathrm{vs}\ \mathrm{NHE}\ \mathrm{at}\ \mathrm{pH}=7\end{array}} $$

5 $$ {\displaystyle \begin{array}{l}{\mathrm{CO}}_2+2{\mathrm{H}}^{+}+2{\mathrm{e}}^{\hbox{-}}\to \mathrm{HCOOH},\\ {}{\mathrm{E}}_0=\hbox{-} 0.61\mathrm{V}\ \mathrm{vs}\ \mathrm{NHE}\ \mathrm{at}\ \mathrm{pH}=7\end{array}} $$

6 $$ {\displaystyle \begin{array}{l}{\mathrm{CO}}_2+2{\mathrm{H}}^{+}+2{\mathrm{e}}^{\hbox{-}}\to \mathrm{CO}+{\mathrm{H}}_2\mathrm{O},\\ {}{\mathrm{E}}_0=\hbox{-} 0.53\mathrm{V}\ \mathrm{vs}\ \mathrm{NHE}\ \mathrm{at}\ \mathrm{pH}=7\end{array}} $$

7 $$ {\displaystyle \begin{array}{l}{\mathrm{CO}}_2+4{\mathrm{H}}^{+}+4{\mathrm{e}}^{\hbox{-}}\to \mathrm{HCHO}+{\mathrm{H}}_2\mathrm{O},\\ {}{\mathrm{E}}_0=\hbox{-} 0.48\mathrm{V}\ \mathrm{vs}\ \mathrm{NHE}\ \mathrm{at}\ \mathrm{pH}=7\end{array}} $$

8 $$ {\displaystyle \begin{array}{l}{\mathrm{CO}}_2+6{\mathrm{H}}^{+}+6{\mathrm{e}}^{\hbox{-}}\to {\mathrm{CH}}_3\mathrm{OH}+{\mathrm{H}}_2\mathrm{O},\\ {}{\mathrm{E}}_0=\hbox{-} 0.38\mathrm{V}\ \mathrm{vs}\ \mathrm{NHE}\ \mathrm{at}\ \mathrm{pH}=7\end{array}} $$

9 $$ {\displaystyle \begin{array}{l}{\mathrm{CO}}_2+8{\mathrm{H}}^{+}+8{\mathrm{e}}^{\hbox{-}}\to {\mathrm{CH}}_4+2{\mathrm{H}}_2\mathrm{O}\\ {}{\mathrm{E}}_0=\hbox{-} 0.24\mathrm{V}\ \mathrm{vs}\ \mathrm{NHE}\ \mathrm{at}\ \mathrm{pH}=7\end{array}} $$

The final products of the photocatalytic-reduction reaction can be the viable test to confirm that the heterojunction photocatalyst was successfully formed.

For example, Chao et al. [61] reported the photocatalytic reduction of CO_2_ under BiOI/g-C_3_N_4_. In their report, photoreduction of CO_2_ to CO and CH_4_ was possible due to high electronegativity of the CB of the as-prepared composite, CO_2_/CO (− 0.53 V) and CO_2_/CH_4_ (− 0.24 V). But the photoreduction of CO_2_ to CH_4_ needs more illumination time to generate more electrons and increase the electron density on CB of BiOI.

## Photocatalytic Applications of Heterostructure g-C3N4

### Pollutant Degradation

The change of human life style is causing thousands of both organic and inorganic pollutants enter the air, water, and soil. Pollutants such as pesticides, industrial chemicals, pharmaceutical chemicals, and heavy metals are common pollutants in the environment [62–68]. These pollutants can be detrimental to the environment and human health [69]. To eliminate these pollutants, different technologies have been employed/involved. These technologies include biological degradation, physical adsorption, filtration, and photocatalytic degradation [70]. Due to its ability to utilize sustainable solar energy for degradation of organic pollutants without causing any side effects to the environment, semiconductor-based photocatalytic degradation has captured the substantial attention [71]. Several semiconductors have been synthesized for the degradation of organic pollutants [7]. For decades, TiO_2_ has emerged as the most common researched semiconductor for several organic pollutant degradation due to its photocatalytic properties, hydrophilicity, high reactivity, reduced toxicity, chemical stability, and lower costs [72]. Recently, graphitic carbon nitride has been the most scientific researched semiconductor due to its narrow band gap of 2.7 eV which permits it to absorb visible light directly without modification. Graphitic carbon nitride (g-C_3_N_4_ ) exhibits high thermal and chemical stability, owing to its tri-s-triazine ring structure and high degree of condensation [24] Although various graphitic carbon nitride semiconductors have been studied for photocatalytic degradation of pollutants, their photocatalytic performance remains unsatisfactory suffering highly from charge (electron–holes) recombination. To overcome the electron–hole recombination in a single g-C_3_N_4_ semiconductor, different researchers have made enormous efforts toward developing novel photocatalytic systems with high photocatalytic activities [73]. The development of heterostructured graphitic carbon nitride photocatalysts semiconductors has proven to be potential for use in enhancing the efficiency of photocatalytic pollutant degradation through the promotion of the separation of photogenerated electron–hole pairs and maximizing the redox potential of the photocatalytic system [59].

For instance, Haiping Li and coworkers reported the solvothermal synthesis of g-C_3_N_4_/Bi_2_MoO_6_ heterostructure with enhanced visible light photocatalytic activity for degradation of rhodamine B (RhB) pollutants in aqueous solution using 1.829 g of as-prepared g-C_3_N_4_ which was added to 0.3234 g of Bi(NO_3_)_3_·5H_2_O in 10 mL of ethylene glycol followed by sonication for 30 min before the addition of 0.0806g of Na_2_MoO_4_·2H_2_O and stirred for 1 h. Using ethylenediamine, the pH was maintained to 7.0 throughout the reaction. The dispersion was heated in the polytetrafluoroethylene-lined stainless autoclave at 160 °C for 6 h and then allowed to cool to room temperature. The solid product was collected by filtration, washed thoroughly with water and ethanol, and dried at 80 °C before it undergone calcination at 400 °C for 1 h to eliminate remained organic species [26].

In their findings, they reported that the photocatalytic activity of g-C_3_N_4_/Bi_2_MoO_6_ (A8) was higher than those of g-C_3_N_4_ and Bi2MoO6, where about 98% of RhB was removed by g-C_3_N_4_/Bi_2_MoO_6_ composite, while less than < 60% was removed by pure g-C_3_N_4_ (A0) or Bi_2_MoO_6_ as seen in Fig. 8a, b. When the experimental data were fitted in a pseudo-first order model (−ln(C/C0) = kt) to quantify the reaction kinetic of photocatalytic RhB degradation, the heterojunction g-C_3_N_4_/Bi_2_MoO_6_ (A8) exhibited the maximum *k* value (0.046 min^−1^) which was three times more than those of g-C_3_N_4_ (A0) or Bi_2_MoO_6_ (A100). This still proves that the heterojunction g-C_3_N_4_/Bi_2_MoO_6_ has high ability to degrade dye pollutants in aqueous than g-C_3_N_4_ and Bi_2_MoO_6_.

Furthermore, Lingjun Song and coworkers reported the facile hydrothermal synthesis of novel g-C_3_N_4_/BiOCl heterostructure nanodiscs for efficient visible light photodegradation of rhodamine B. In the heterostructure composite synthesis, a well-dispersed suspension of protonated g-C_3_N_4_ was prepared by dissolving a portion of the as-prepared g-C_3_N_4_ in 6.5 mL of hydrochloric acid under magnetic stirring followed by subsequently addition of 5 mmol of Bi(NO_3_)_3_. 5H2O, KCl, and deionized (DI) water (15 mL). The pH of the mixture was subsequently adjusted to 6 with dilute NaOH solution. The white suspension obtained after continuous vigorous stirring for 2 h was heated at 140 °C for 12 h and allowed to cool to room temperature. The precipitates were collected by centrifugation, thoroughly washed with DI water and dried at 80 °C in air to furnish the target sample [76]_._ The effective separation of photogenerated electron–hole pairs, due to the charge transfer at the interface between two types of semiconductors in the composite, increased the photocatalytic activity of g-C_3_N_4_/BiOCl (95%) than that of individual g-C3N4 (30%) and BiOCl (52%).

**Fig. 9:**
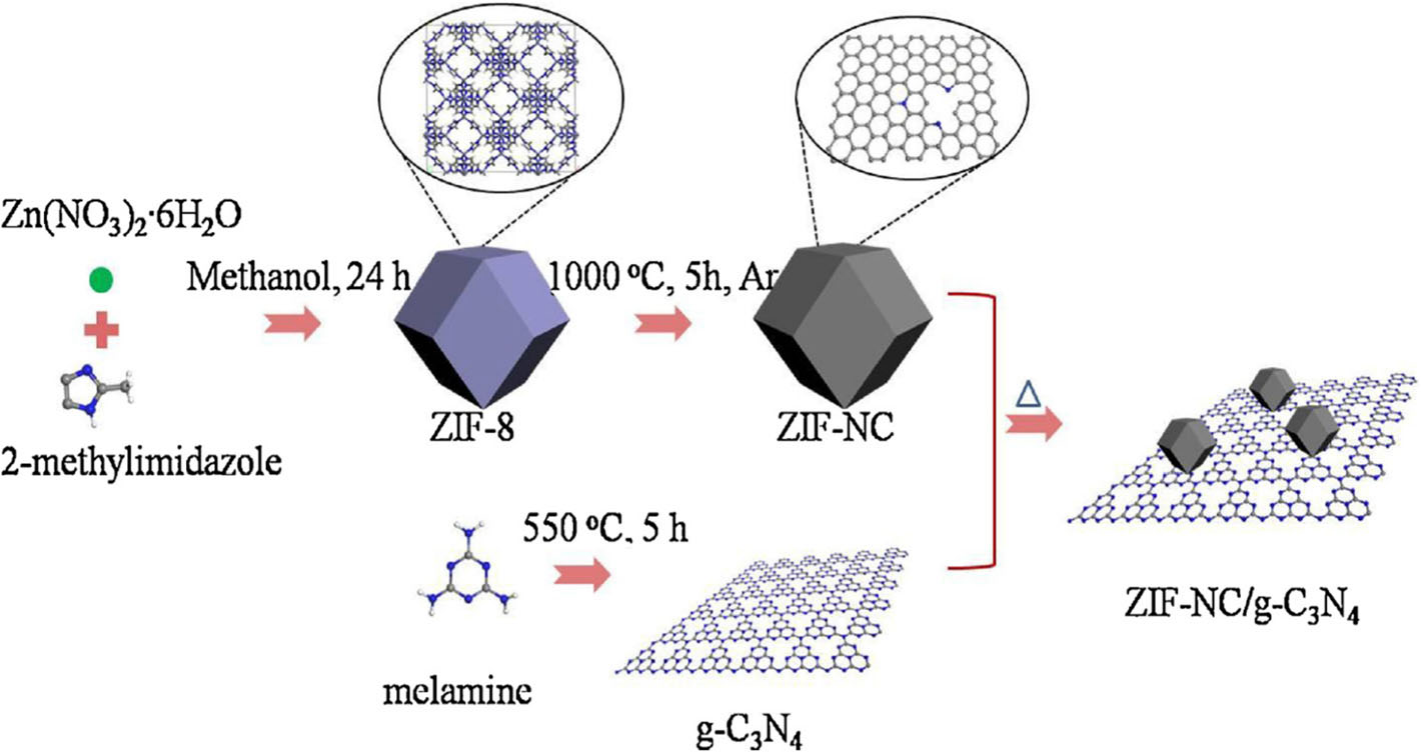
Schematic illustration of the formation of ZIF-NC/g-C_3_N_4_ composite. Reproduced with permission [24]. Copyright 2018 Elsevier B.V

Yan Gong and coworkers reported the synthesis of the novel metal organic framework (ZIF-8)-derived nitrogen-doped carbon (ZIF-NC) modified g-C3N4-heterostructured composite by the facile thermal treatment method where an appropriate amount of ZIF-CN in a methanol solution was firstly placed in an ultrasonic bath for 30 min to completely disperse the ZIF-NC before g-C_3_N_4_ powder was added and stirred for 24 h. After volatilization of the methanol in water bath at 60° C, the obtained powder was heated to 300° C for 2 h under atmosphere (Fig. 9). In their report, photocatalytic activity of ZIF-NC/g-C_3_N_4_ for the degradation of bisphenol A (BPA) in aqueous solution reached the removal rate of 97% after 60 min of irradiation with 0.5% ZIF-NC content. Excessive addition of the ZIN-NC to 1% over g-C_3_N_4_ surfaces hinder the light adsorption of g-C_3_N_4_ which results in low generation of electron–hole pairs on g-C_3_N_4_, hence resulting to decreased photocatalytic activity [58].

**Fig. 10:**
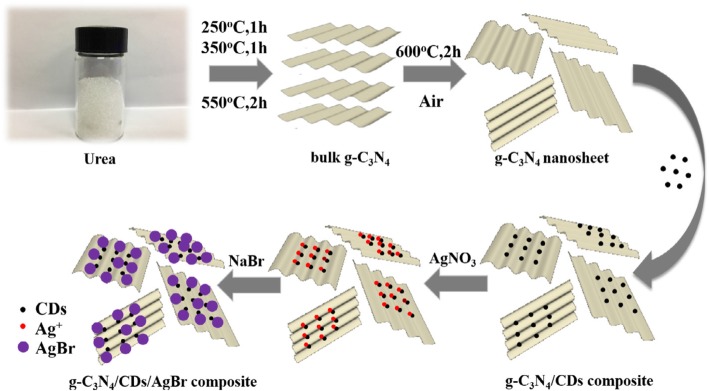
Schematic illustration of preparation process of the g-C_3_N_4_/CDs/AgBr nanocomposite. Reproduced with permission [86]. Copyright 2017 Elsevier B.V

Xuli Miao and coworkers synthesized g-C_3_N_4_/AgBr nanocomposite decorated with carbon dots as a highly efficient visible light-driven photocatalyst by introduction of carbon dots (CDs) onto the surface of g-C_3_N_4_, followed by in-situ growth of AgBr nanoparticles on CD-modified g-C_3_N_4_ nanosheets (Fig. 10). After the evaluation of as-prepared samples for the degradation of RhB under visible light irradiation, they found that the ternary composites of g-C_3_N_4_/CDs/AgBr show higher photocatalytic activity than single AgBr, g-C_3_N_4_ with the RhB degradation rate reaching 96% after 40 min of irradiation [105].

Jiajia Wang and his coworkers reported the synthesis of Atomic scale g-C_3_N_4_/Bi_2_WO_6_ 2D/2D heterojunction with enhanced photocatalytic degradation of ibuprofen (IBF) under visible light irradiation. As reported, as-prepared atomic scale g-C_3_N_4_ showed high photocatalytic activity (∼ 96.1%) compared to that of pure g-C_3_N_4_ (38.2%) and of pure m-B_2_WO_6_ (67.3%) under the same experimental conditions. This also proves that there was high separation of photogenerated charge carriers in atomic scale g-C_3_N_4_/Bi_2_WO_6_ 2D/2D heterojunction thus enhancing photocatalytic degradation efficiency of IBF.

Several other researches on the photocatalytic activities of the g-C3N4 heterojunction performances have been conducted by different researchers on different pollutants as summarised in Table 1.

**Table 1:** Studies of g-C_3_N_4_ heterojunctions for various pollutants degradations

Photocatalyst	Light source	Application	Performance	Ref.
g-C_3_N_4_/Bi_2_MoO_6_	300-W Xenon lamp (*λ* > 420-nm cut-off filter)	RhB degradation	*k* = 6.484 × 10^−2^ min^−1^	[74]
g-C_3_N_4_/Bi_2_MoO_6_	50-W 410-nm LED light	Methylene blue (MB) degradation	*k* = 6.88 × 10^−2^ min^−1^	[75]
g-C_3_N_4_/RGO/Bi_2_MoO_6_	500-W Xenon lamp (*λ* > 420-nm cut-off filter)	RhB degradation	*k* = 5.5 × 10^−2^ min^−1^	[45]
Bi_2_MoO_6_/CNTs/g-C_3_N_4_	500-W Xenon lamp (*λ* > 420-nm cut-off filter)	2,4-Dibromophenol debromination and degradation	k = 7.8 × 10^−3^ min^−1^	[46]
g-C_3_N_4_/BiOCl	300-W Xenon arc lamp (*λ* ≥ 400-nm cut-off filter)	RhB degradation	*k* = 1.99 × 10^−1^ min^−1^	[76]
g-C_3_N_4_/Bi_2_MoO_6_	400-W Metal halide lamp (*λ* ≥ 420-nm cut-off filter)	RhB degradation	*k* = 4.6 × 10^−2^ min^−1^	[26]
Bi_2_O_3_/g-C_3_N_4_	300-W Xenon lamp (*λ* > 400-nm cut-off filter)	Phenol degradation	*k* = 2.47 × 10^−3^ min^−1^	[77]
Bi_2_O_3_/g-C_3_N_4_	500-W Xenon lamp (*λ* > 400-nm cut-off filter)	RhB degradation Methylene blue degradation	*k* = 2.53 × 10^−2^ min^−1^ *k* = 1.01 × 10^−2^ min^−1^	[78]
BiVO_4_/g-C_3_N_4_	PLS-SXE300 Xenon lamp	RhB degradation	*k* = 9.307 × 10^−2^ min^−1^	[79]
Bi_2_O_3_/g-C_3_N_4_	35-W Xenon lamp	Amido black 10B degradation	*k* = 1.722 × 10^−2^ min^−1^	[80]
Bi_2_O_3_/g-C_3_N_4_	300-W Xenon lamp (*λ* > 420nm cut-off filter)	Methylene blue degradation	*k* = 6.3 × 10^−^ min^−1^	[81]
WO_3_/g-C_3_N_4_	500-W Xenon lamp (*λ* > 400nm cut-off filter)	Methylene blue degradation Fuchsin (BF) degradation	*k* = 3.53 × 10^−2^ min^−1^ *k* = 2.38 × 10^−2^ min^−1^	[82]
500-W Xenon lamp	500-W Xenon lamp	RhB degradation	*k* = 1.08 × 10^−2^ min^−1^	[83]
WO_3_/g-C_3_N_4_	300-W Xenon lamp (*λ* > 400-nm cut-off)	Methylene blue degradation	*k* = 1.3933h^−1^	[84]
g-C_3_N_4_/MoO_3_	300-W Xenon lamp (*λ* > 400nm)	Methylene blue degradation	*k* = 8.837 × 10^−1^h^−1^	[85]
β-Bi_2_O_3_/g-C_3_N_4_	150-W Xenon lamp (420-nm cut-off filter)	Methylene blue degradation	*k* = 1.727 × 10^−2^ min^−1^	[86]
g-C_3_N_4_/Bi_2_WO_6_	300-W Xenon lamp (350–780-nm cut-off filter)	2,4-dichlorophenol dechlorination (2,4-DCP)	*k* = 1.13 h^−1^	[87]
Ag_3_PO_4_/g-C_3_N_4_	300-W Xenon arc lamp (420-nm cut-off filter)	*k* = 1.158 × 10^−1^ min^−1^	*k* = 1.158 × 10^−1^ min^−1^	[88]
MoO_3_/g-C_3_N_4_	350-W Xenon lamp (420-nm cut-off filter)	Tetracycline degradation	*k* = 2.31 × 10^−2^ min^−1^	[47]
BiVO_4_/g-C_3_N_4_	500-W Xenon lamp (*λ* > 420-nm cut-off filter)	RhB degradation	*k* = 3.42 × 10^−1^ h^−1^	[89]
WO_3_/g-C_3_N_4_	300-W Xenon lamp (420-nm cut-off filter)	Ceftiofur sodium (CFS) degradation Tetracycline hydrochloride (TC-HCl) degradation	*k* = 1.64 × 10 ^2^ min^−1^ *k* = 1.2 × 10^−2^ min^−1^	[90]
g-C_3_N_4_/TiO_2_	15 W, 365-nm UV lamp	Formaldehyde (HCHO) degradation	*k* = 7.36 × 10^−2^ min^−1^	[91]
B_i2_O_3_/g-C_3_N_4_	300-W Xenon lamp(*λ* > 420nm)	RhB degradation	*k* = 7.46 × 10^−2^ min^−1^	[92]
g-C_3_N_4_/TiO_2_	3-W 365-nm UV lamp	Brilliant red X3B degradation	*k* = 5.1 × 10^−2^ min^−1^	[93]
Bi_2_O_3_/g-C_3_N_4_	500-W Xenon arc lamp (400-nm cut-off filter)	RhB degradation	*k* = 1.01 × 10^−2^ min^−1^	[78]
g-C_3_N_4_/Ag_2_CO_3_	300-W Xenon arc lamp (400-nm cut-off filter)	RhB degradation	*k* = 1.36 × 10^−1^ min^−1^	[94]
g-C_3_N_4_/Bi_5_O_7_I	300-W Xenon lamp (*λ* > 420-nm cut-off filter)	RhB deghradation Methyl orange (MO) degration	*k* = 1.97 × 10^−1^ min^−1^ *k* = 8.4 × 10^−2^ min^−1^	[95]
g-C_3_N_4_/Bi_2_WO_6_	300-W Xenon lamp	Ibuprofen degradation	*k* = 5.2 × 10^−^2 min^−1^	[60]
V_2_O_5_/g-C_3_N_4_	250-W Xenon lamp (420-nm cut-off filter)	RhB degradation	*k* = 4.91 × 10^−2^ min^−1^	[96]
Al_2_O_3_/g-C_3_N_4_	350W Xenon lamp (400-nm cut-off filter)	RhB degradation	*k* = 2.57 × 10^−2^ min^−−1^	[97]
MoS_2_/g-C_3_N_4_	300-W Xenon lamp (*λ* > 420-nm cut-off filter)	RhB degradation Methyl orange degradation	*k* = 1.52 × 10^−^1 min^−1^ *k* = 1.61 × 10^−2^ min^−1^	[98]
CuO/g-C_3_N_4_	300-W Xenon lamp (*λ* > 420-nm cut-off filter)	Salicylic acid degradation	94% degradation	[99]
g-C_3_N_4_-Cu_2_O	LED lamp	Methyl orange degradation	84% degradation	[100]
g-C_3_N_4_/BiOI	Visible light	RhB degradation	*k* = 3.99 × 10^−2^ min^−1^	[101]
g-C_3_N_4_/TiO_2_	30-W visible light lamp	Orange II degradation	*k* = 3.11 × 10^−2^ min^−1^	[102]
Bi_2_MoO_6_/g-C_3_N_4_	300-W Xenon lamp (*λ* > 420-nm cut-off filter)	Bacterial disinfection(*E.Coli* DH5α)	*k* = 1.269h^−1^	[103]
g-C_3_N_4_/CeO_2_	50-W compact fluorescent lamp (*λ* > 400-nm cut-off filter)	Methylene blue degradation	*k* = 2.46 × 10^−1^h^−1^	[104]

### Photocatalytic Hydrogen Gas (H_2_) Production

Depletion of the fossil fuel energy has made the production of hydrogen gas (H_2_) which has high heat energy value to receive much research attention recently [106]. Solar energy convention remains to be the promising technology for water splitting mechanism to generate H_2_ because of its simplicity and clean reactions [107–109]. Different photocatalysts has been studied on the water splitting for the H_2_ production (see Table 2).

**Table 2:** Hydrogen production study by different g-C3N4 heterostructures

Photocatalyst	Source of light	Application	Performance	Ref.
g-C3N4/Au/CdS	300-W Xenon lamp (420-nm cut-off filter)	Hydrogen production	530 μmol after 5 h	[110]
WO3/g-C3N4	Artificial solar light	Hydrogen production	110 μmol h^−1^g^−1^	[107]
C,N-TiO_2_/g-C_3_N_4_	300-W Xenon arc lamp (400-nm cut-off filter)	Hydrogen production	39.18 mmol h^−1^g^−1^	[111]
WO_3_/g-C_3_N_4_	300-W Xenon lamp (*λ* > 420-nm cut-off filter)	Hydrogen production	1853 μmol h^−1^g^−1^	[106]
g-C_3_N_4_/WS_2_	300-W Xenon arc lamp (*λ* ≥ 420-nm cut-off filter)	Hydrogen production	101 μmol h^−1^g^−1^	[112]
Bi_2_MoO_6_/g-C_3_N_4_	300-W Xenon lamp (*λ* > 420nm cut-off filter)	Hydrogen production	563.4 μmol h^−1^g^−1^	[103]

For example, She and coworkers reported the synthesis of 2D α-Fe_2_O_3_/g-C_3_N_4_ Z-scheme catalysts. As reported, H_2_ evolution activity was further enhanced in the hybrids with α-Fe_2_O_3_ nanostructures, reaching 31400 μmol g^−1^ h^−1^ for α-Fe_2_O_3_/2D g-C_3_N_4_ (α-Fe_2_O_3_ loading 3.8 wt.%). Photocurrent experiments also confirmed the higher activity of α-Fe_2_O_3_/2D g-C_3_N_4_ (3.8 wt. %) in comparison with samples containing ML g-C_3_N_4_and α-Fe_2_O_3_ [113]. With these results, it is evident that heterostructured carbon nitride semiconductors have high photocatalytic efficiency on hydrogen production [109]. The photocatalytic hydrogen production by other studies of g-C_3_N_4_ heterojunctions are summarized in Table 2.

The photocatalytic hydrogen (H_2_) production is hampered by the difficulty of separating the hydrogen and oxygen-containing products (hydrogen storage mechanism) which is caused by very close distance between reduction-oxidation sites. This in turn result into difficulties to separately deliver photogenerated electron and holes to the reduction and oxidation site, respectively, in the designed photocatalyst which might cause reverse reaction of hydrogen- and oxygen-containing products or even damages by explosion. In overcoming this challenge, studies have been made on how to feasibly separate produced hydrogen from oxygen-containing products while maintaining the close distance between reduction-oxidation site which is very essential photogenerated charge transfer. In 2017, Li Yang and coworkers synthesised sandwich structures of graphene with combined photocatalytic hydrogen production and storage ability [114]. In their study, the synthesized sandwiched graphene allows the penetration of only proton to the reduction site to produce hydrogen inside the sandwich. This not only to prevent the reverse reaction but also to facilitate the safe storage of the generated hydrogen reaching the storage rate of 5.2 wt% which is very close to the US Department of Energy standards (6.5 wt%). Also, Xijun Wang and coworkers synthesized the carbon–quantum-dot/carbon nitride hybrid with high ability of isolating hydrogen from oxygen in the photocatalytic water splitting using the first-principles calculation [115]. In this study, it was found that only protons were allowed to penetrate the inner layer of graphene to produce H_2_. The produced hydrogen gas was then capsuled in the inner layer of the synthesised photocatalyst. This also prevents the reverse reaction and makes the availability of the produced hydrogen (H_2_).

### CO_2_ Reduction

The population growth and industrialization has been detrimental the environment including the atmosphere [116]. CO_2_ increase recently has remained to be the crucial agenda in the universe [117, 118]. CO_2_ produced from burning of fuel from domestic to industrial level has contributed much on the atmospheric air pollution hence resulting into the current global warming the world is suffering today [119–121]. Different strategies have been developed to cut down the production of CO_2_. The SDG 7 pinpoint for the clean and renewable energy as one way of reducing the production of CO_2_ in the atmosphere [122, 123]. But increasing demand of fuel and productions in the industries still make the contribution of CO_2_ to be high (Table 3). Technologies have been developed to degrade the produced CO_2_. Among others, photocatalytic reactions have promised to be one of the best technologies for the CO_2_ reduction.

**Table 3:** Studies of g-C_3_N_4_ heterojunctions on Carbon dioxide (CO_2_) reduction

Photocatalyst	Source of light	Application	Performance	Ref.
g-C_3_N_4_/ZnO	300-W Xenon arc lamp	CO_2_ reduction	0.6 μmol h^−1^g^−1^ CH_3_OH	[124]
SnO_2-X_/g-C_3_N_4_	500-W Xenon lamp	CO_2_ reduction	22.7 μmol h^−1^ g^−1^ CO, CH_3_OH, CH_4_	[125]
BiOI/g-C_3_N_4_	300-W Xenon arc lamp (*λ* > 400-nm cut-off filter)	CO_2_ reduction	17.9 μmol g^−1^ CO	[61]

Sheng Zhou and coworkers reported the facile in situ synthesis of graphitic carbon nitride (g-C_3_N_4_)-N-TiO_2_ heterojunction as an efficient photocatalyst for the selective photoreduction of CO_2_ to CO. The composites of graphitic carbon nitride and nitrogen-doped titanium dioxide composites (g-C_3_N_4_-N-TiO_2_) were in situ synthesized by thermal treatment of the well-mixed urea and Ti(OH)_4_ in an alumina crucible with a cover at different mass ratios. The mixture was heated to 550° C for 3 h and then 580° C for 3 h at a heating rate of 5° C min^−1^ to obtain the product. The product was washed with nitric acid (0.1 M) and distilled water for several times to remove residual alkaline and sulfate species (e.g., ammonia and SO_4_^2−^) adsorbed on the sample, and then dried at 80° C overnight to get the final product.

In their report, photocatalytic of CO_2_ reduction was carried out in a gas-closed circulation system operated under simulated light irradiation with photocatalyst, CO_2_, and water vapor sealed in the system. The heterojunction between g-C_3_N_4_ and nitrogen-doped TiO_2_ demonstrated enhanced catalytic performance reaching the highest CO evolution amount (14.73 μmol) during light irradiation compared with P25 (3.19 μmol) and g-C_3_N_4_ (4.20 μmol) samples. The heterojunction between g-C_3_N_4_ and nitrogen-doped TiO_2_ showed the high activity because it promotes the separation of light-induced electrons and holes. These results prove that the heretostructured carbon nitride semiconductor has high photocatalytic CO_2_ reduction as compared to their precursors [126]. More studies on the heterojunctions of g-C3N4 for photocatalytic reduction of CO_2_ are summarized in Table 3.

## Photocatalyst Stability

The stability of photocatalysts is crucial for their practical application [59]. It shows how the photocatalysts can be reused without or with little loss in their activities [21]. In order to know the reusability of the photocatalyst, the degradation of the pollutant by the same composite for several times/cycles are performed [127]

**Fig. 11:**
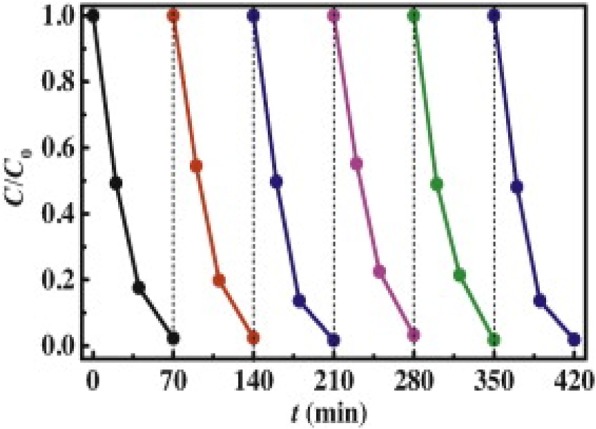
Cycling runs for photocatalytic degradation of RhB over g-C_3_N_4_/Bi_2_MoO_6_ composite A8 under visible light irradiation. Reproduced with permission [23]. Copyright 2014 Elsevier B.V

The as-synthesized g-C_3_N_4_/Bi_2_MoO_6_ heterojunction photocatalyst exhibited excellent stability in the visible light photochemical degradation reactions. Figure 11 shows that after six consecutive runs, no apparent deactivation of the composite g-C_3_N_4_/Bi_2_MoO_6_ (A8) is observed, and the RhB degradation efficiency declines by < 1%.

Wang and coworkers [115] then designed a hybrid structure of carbon-quantum-dots (CQDs) attaching to a single-layered carbon nitride (C3N) material. These scientists showed that the hybrid can harvest visible and infrared light for water splitting. Also, Darkwah and Ao also discussed how stable the carbon nitride can work more efficiently in degradation of both organic and inorganic compounds for wastewater treatment and related applications [22, 128, 129].

### Future Viewpoint of Heterostructure g-C_3_N_4_

The future research of heterostructure g-C3N4 nano-based photocatalyst may focus on the design and synthesis of more effective nanostructures, which are responsive to morphology monitoring, evaluating the photocatalysis practicality, and the degradation behavior and mechanism of more types of pollutants, especially for non-dyed pollutants and then exploring the applications of diverse g-C3N4 nano-based particles in treating wastewater, its effective application in solar energy utilization, sensing applications by fully assessing their photocatalytic ability, cost, energy consumption, and reusability.

One of the key areas to consider for future studies should mainly focus on employing new technologies or combination of the existing techniques of increasing the settling velocity of g-C3N4 to upturn the run-off rate that could be used to improve the material for improving photocatalytic activities.

## Conclusion

Although photocatalytic degradation is an ideal strategy for cleaning environmental pollution, it remains challenging to construct a highly efficient photocatalytic system by steering the charge flow in a precise manner. Different researches have proven the high photocatalytic activity of the heterostructured semiconductors over pollutants degradation, hydrogen gas evolution, and carbon dioxide reduction. Among others, heterostructured carbon nitride (CN) semiconductors in recent decades have shown the anonymous photocatalytic activity towards organic pollutants, hydrogen production, and carbon dioxide. Reasonably, g-C_3_N_4_ has revealed to be one of the best candidates suitable for developing and assembling state-of-the-art composite photocatalysts. Therefore, there is slight doubt that the considerable advancement of g-C_3_N_4_ nano-based particle will endure to develop in the near future. Hence, more researches should consider its modification structures, mechanisms, and the degradative abilities of this candidate

## Data Availability

Not applicable

## Abbreviations

g-C_3_N_4_: Graphite carbon nitride
TiO_2_: Titanium oxide
ZnO: Zinc oxide

## References

[1] Hoffmann MR, Martin ST, Choi W, Bahnemann DW (1995). Environmental applications of semiconductor photocatalysis. Chem Rev..

[2] Tong H, Ouyang S, Bi Y, Umezawa N, Oshikiri M, Ye J (2012). Nano-photocatalytic materials: possibilities and challenges. Adv Mater..

[3] Low J, Jiang C, Cheng B, Wageh S, Al-Ghamdi AA, Yu J (2017). A review of direct Z-scheme photocatalysts. Small Methods.

[4] Yuan Y (2017). Construction of g-C3N4/CeO2/ZnO ternary photocatalysts with enhanced photocatalytic performance. J Phys Chem Solids.

[5] Umar M, Abdul H (2013) Photocatalytic degradation of organic pollutants in water. Org. Pollut. - Monit. Risk Treat

[6] Felis E, Sochacki A, Magiera S (2016). Degradation of benzotriazole and benzothiazole in treatment wetlands and by artificial sunlight. Water Res..

[7] Yan P (2015). Photovoltaic device based on TiO2 rutile/anatase phase junctions fabricated in coaxial nanorod arrays. Nano Energy.

[8] Xing Z (2018). Recent advances in floating TiO2-based photocatalysts for environmental application. Appl Catal B Environ.

[9] Huang Y, Zhang W, Zhang M, Zhang X, Zhao Y (2018). Hydroxyl-functionalized TiO2@SiO2@Ni/nZVI nanocomposites fabrication, characterization and enhanced simultaneous visible light photocatalytic oxidation and adsorption of arsenite. Chem Eng J.

[10] Pan J, Dong Z, Wang B, Jiang Z, Zhao C, Wang J (2019). The enhancement of photocatalytic hydrogen production via Ti 3 + self- doping black TiO 2 / g-C 3 N 4 hollow core-shell nano-heterojunction. Appl Catal B Environ.

[11] Vaiano V, Iervolino G, Rizzo L (2018). Cu-doped ZnO as efficient photocatalyst for the oxidation of arsenite to arsenate under visible light. Appl Catal B Environ.

[12] Chen L, He J, Liu Y, Chen P, Au CT, Yin SF (2016). Recent advances in bismuth-containing photocatalysts with heterojunctions. Chinese J Catal..

[13] Amaranatha Reddy TKKD, Park H, Ma R, Kumar DP, Lim M (2009). Supporting Information.

[14] Fu J, Yu J, Jiang C, Cheng B (2018). g-C3N4-Based heterostructured photocatalysts. Adv Energy Mater..

[15] Liang J, Liu F, Deng J, Li M, Tong M (2017). Efficient bacterial inactivation with Z-scheme AgI / Bi 2 MoO 6 under visible light irradiation. Water Res..

[16] Suyana P, Ganguly P, Nair BN, Mohamed AP, Warrier KGK, Hareesh US (2017). Co3O4-C3N4p-n nano-heterojunctions for the simultaneous degradation of a mixture of pollutants under solar irradiation. Environ Sci Nano.

[17] Zhang X, Ke X, Yao J (2018). Recent development of plasmon-mediated photocatalysts and their potential in selectivity regulation. J Mater Chem A.

[18] Qin J, Huo J, Zhang P, Zeng J, Wang T, Zeng H (2016). Improving the photocatalytic hydrogen production of Ag/g-C3N4nanocomposites by dye-sensitization under visible light irradiation. Nanoscale.

[19] Atabaev TS (2018). Plasmon-enhanced solar water splitting with metal oxide nanostructures: a brief overview of recent trends. Front Mater Sci..

[20] Song H, Zhang L, Su Y, Lv Y (2017) Recent advances in graphitic carbon nitride-based chemiluminescence, cataluminescence and electrochemiluminescence. J Anal Test*.*:274–290

[21] Dong H, Guo X, Yang C, Ouyang Z (2018). Synthesis of g-C3N4by different precursors under burning explosion effect and its photocatalytic degradation for tylosin. Appl Catal B Environ.

[22] Darkwah WK, Ao Y (2018). Mini review on the structure and properties ( photocatalysis ), and preparation techniques of graphitic carbon nitride nano-based particle , and its applications. Nano Res Lett.

[23] Liu J, Zhang T, Wang Z, Dawson G, Chen W (2011). Simple pyrolysis of urea into graphitic carbon nitride with recyclable adsorption and photocatalytic activity. J Mater Chem..

[24] Tian L (2018). Molten salt synthesis of tetragonal carbon nitride hollow tubes and their application for removal of pollutants from wastewater. Appl Catal B Environ.

[25] Kanagaraj T, Thiripuranthagan S (2017). Visible light photocatalytic activities of template free porous graphitic carbon nitride — BiOBr composite catalysts towards the mineralization of reactive dyes. Appl Surf Sci..

[26] Li H, Liu J, Hou W, Du N, Zhang R, Tao X (2014). Synthesis and characterization of g-C3N4/Bi2MoO6 heterojunctions with enhanced visible light photocatalytic activity. Appl Catal B Environ..

[27] F. K. Kessler et al., “Functional carbon nitride materials-design strategies for electrochemical devices,” Nat Rev Mater*.*, 2, no. May, 2017.

[28] Zhou Z, Zhang Y, Shen Y, Liu S, Zhang Y (2018). Molecular engineering of polymeric carbon nitride: advancing applications from photocatalysis to biosensing and more. Chem Soc Rev..

[29] Groenewolt M, Antonietti M (2005). Synthesis of g-C3N4 nanoparticles in mesoporous silica host matrices. Adv Mater..

[30] Goettmann F, Fischer A, Antonietti M, Thomas A (2006). Chemical synthesis of mesoporous carbon nitrides using hard templates and their use as a metal-free catalyst for Friedel-Crafts reaction of benzene. Angew Chemie Int Ed..

[31] Gong Y (2018). MOF-derived nitrogen doped carbon modified g-C 3 N 4 heterostructure composite with enhanced photocatalytic activity for bisphenol A degradation with peroxymonosulfate under visible light irradiation. Appl Catal B Environ.

[32] Li H, Zhou Y, Tu W, Ye J, Zou Z (2015). State-of-the-art progress in diverse heterostructured photocatalysts toward promoting photocatalytic performance. Adv Funct. Mater..

[33] Jiang L (2017). Doping of graphitic carbon nitride for photocatalysis: a reveiw. Appl Catal B Environ..

[34] Yan W, Yan L, Jing C (2019). Impact of doped metals on urea-derived g-C3N4 for photocatalytic degradation of antibiotics: structure, photoactivity and degradation mechanisms. Appl Catal B Environ.

[35] Xu Y (2019). One-step synthesis of Fe-doped surface-alkalinized g-C3N4 and their improved visible-light photocatalytic performance. Appl Surf Sci.

[36] Jiang L (2018). Nitrogen self-doped g-C3N4 nanosheets with tunable band structures for enhanced photocatalytic tetracycline degradation. J Colloid Interface Sci..

[37] Ling F, Li W, Ye L (2019). The synergistic effect of non-metal doping or defect engineering and interface coupling on the photocatalytic property of g-C3N4: First-principle investigations. Appl Surf Sci.

[38] Guo W, Zhang J, Li G, Xu C (2019). Enhanced photocatalytic activity of P-type (K, Fe) co-doped g-C3N4 synthesized in self-generated NH3 atmosphere. Appl Surf Sci.

[39] Fan J, Qin H, Jiang S (2019). Mn-doped g-C3N4 composite to activate peroxymonosulfate for acetaminophen degradation: the role of superoxide anion and singlet oxygen. Chem Eng J.

[40] Xie M (2018). Cobalt doped g-C3N4 activation of peroxymonosulfate for monochlorophenols degradation. Chem Eng J.

[41] Zhu Z (2019). Insight into the effect of co-doped to the photocatalytic performance and electronic structure of g-C3N4 by first principle. Appl Catal B Environ.

[42] Wu W et al (2019) In situ preparation and analysis of bimetal Co-doped mesoporous graphitic carbon nitride with enhanced photocatalytic activity. Nano-Micro Lett 11(1) 10.1007/s40820-018-0236-y PMC7770843 34137960

[43] Shu Z (2019). A green one-pot approach for mesoporous g-C3N4 nanosheets with in situ sodium doping for enhanced photocatalytic hydrogen evolution. Int J Hydrogen Energy.

[44] Wang Q, Han XH, Sommers A, Park Y, T’Joen C, Jacobi A (2012). A review on application of carbonaceous materials and carbon matrix composites for heat exchangers and heat sinks. Int J Refrig..

[45] D. Ma, J. Wu, M. Gao, Y. Xin, Y. Sun, T. Ma, “Hydrothermal synthesis of an artificial Z-scheme visible light photocatalytic system using reduced graphene oxide as the electron mediator,” Chem Eng J*.*, 2016.

[46] Ma D, Wu J, Gao M, Xin Y, Chai C (2017). Enhanced debromination and degradation of 2 , 4-dibromophenol by an Z-scheme Bi2MoO6/CNTs/g-C3N4 visible light photocatalyst. Chem Eng J..

[47] Xie Z (2018). Construction of carbon dots modified MoO3/g-C3N4Z-scheme photocatalyst with enhanced visible-light photocatalytic activity for the degradation of tetracycline. Appl Catal B Environ.

[48] Xia J (2017). Facile fabrication of g-C3N4/BiPO4 hybrid materials via a reactable ionic liquid for the photocatalytic degradation of antibiotic ciprofloxacin. J Photochem Photobiol A Chem..

[49] Reza Gholipour M, Dinh C-T, Béland F, Do T-O (2015). Nanocomposite heterojunctions as sunlight-driven photocatalysts for hydrogen production from water splitting. Nanoscale.

[50] Opoku F, Govender KK, van Sittert CGCE, Govender PP (2018). Insights into the photocatalytic mechanism of mediator-free direct Z-scheme g-C3N4/Bi2MoO6(010) and g-C3N4/Bi2WO6(010) heterostructures: a hybrid density functional theory study. Appl Surf Sci..

[51] Marschall R (2014). Semiconductor composites: strategies for enhancing charge carrier separation to improve photocatalytic activity. Adv Funct Mater..

[52] Wei Z (2019). Photocatalytic hydrogen evolution with simultaneous antibiotic wastewater degradation via the visible-light-responsive bismuth spheres-g-C 3 N 4 nanohybrid : waste to energy insight. Chem Eng J.

[53] Faisal M, Ismail AA, Harraz FA, Al-sayari SA (2018). Fabrication of highly effi cient TiO 2 / C 3 N 4 visible light driven photocatalysts with enhanced photocatalytic activity. J Mol Struct..

[54] Eslami H (2018). Efficient photocatalytic oxidation of arsenite from contaminated water by Fe2O3-Mn2O3nanocomposite under UVA radiation and process optimization with experimental design. Chemosphere.

[55] Litter MI, Levy IK (2014). TiO 2 -photocatalytic reduction of pentavalent and trivalent arsenic : production of elemental arsenic and arsine. The 5th International Congress on Arsenic in the Environment,May 11-16, 2014.

[56] Guo Y, Zhang L, Zhou K, Shen Y, Zhang Q, Gu C (2014). Selective gold recovery by carbon nitride through photoreduction. J Mater Chem A.

[57] Ao Y, Bao J, Wang P, Wang C (2017). A novel heterostructured plasmonic photocatalyst with high photocatalytic activity: Ag@AgCl nanoparticles modified titanium phosphate nanoplates. J Alloys Compd..

[58] Ye F, Li H, Yu H, Chen S, Quan X (2018). Constructing BiVO4-Au@CdS photocatalyst with energic charge-carrier-separation capacity derived from facet induction and Z-scheme bridge for degradation of organic pollutants. Appl Catal B Environ.

[59] Wan Z, Zhang G, Wu X, Yin S (2017). Novel visible-light-driven Z-scheme Bi12GeO20/g-C3N4photocatalyst: oxygen-induced pathway of organic pollutants degradation and proton assisted electron transfer mechanism of Cr(VI) reduction. Appl Catal B Environ..

[60] Wang J (2017). Atomic scale g-C3N4/Bi2WO62D/2D heterojunction with enhanced photocatalytic degradation of ibuprofen under visible light irradiation. Appl Catal B Environ..

[61] Wang JC (2016). Indirect Z-Scheme BiOI/g-C3N4 photocatalysts with enhanced photoreduction CO2 activity under visible light irradiation. ACS Appl. Mater Interfaces.

[62] Oliveira TS, Murphy M, Mendola N, Wong V, Carlson D, Waring L (2015). Characterization of pharmaceuticals and personal care products in hospital effluent and waste water influent / effluent by direct-injection LC-MS-MS. Sci Total Environ..

[63] Komorowicz I, Barałkiewicz D (2016) Determination of total arsenic and arsenic species in drinking water, surface water, wastewater, and snow from Wielkopolska, Kujawy-Pomerania, and Lower Silesia provinces, Poland. Environ Monit Assess 188(9) 10.1007/s10661-016-5477-y PMC4972851 27488197

[64] Morita K, Kaneko E (2006). Spectrophotometric determination of arsenic in water samples based on micro particle formation of ethyl violet-molybdoarsenate. Anal Sci..

[65] Zaharin A, Soraya A, Mangala S (2014). Occurrence of 17 α -ethynylestradiol ( EE2 ) in the environment and effect on exposed biota : a review. Environ Int..

[66] Nava JL, Quiroz MA, Martínez-Huitle CA (2008). Electrochemical treatment of synthetic wastewaters containing alphazurine a dye: Role of electrode material in the colour and COD removal. J Mex. Chem Soc..

[67] Borowska E, Felis E, Kalka J (2016). Oxidation of benzotriazole and benzothiazole in photochemical processes: kinetics and formation of transformation products. Chem Eng J..

[68] Nthunya LN, Masheane ML, Malinga SP, Nxumalo EN, Mamba BB, Mhlanga SD (2017). Determination of toxic metals in drinking water sources in the Chief Albert Luthuli Local Municipality in Mpumalanga, South Africa. Phys Chem Earth.

[69] Song S, Lu C, Wu X, Jiang S, Sun C, Le Z (2018). Strong base g-C3N4with perfect structure for photocatalytically eliminating formaldehyde under visible-light irradiation. Appl Catal B Environ.

[70] Han A, Zhang H, Lu D, Sun J, Chuah GK, Jaenicke S (2018). Efficient photodegradation of chlorophenols by BiOBr/NaBiO3heterojunctioned composites under visible light. J Hazard Mater..

[71] Yang JC (2011). Developing an iron-carbon nitride complex as photocatalyst with response to visible light. Adv Mater Res..

[72] Li X, Yu J, Low J, Fang Y, Xiao J, Chen X (2015). Engineering heterogeneous semiconductors for solar water splitting. J Mater Chem A.

[73] Akhundi A, Habibi-Yangjeh A (2015). Ternary g-C3N4/ZnO/AgCl nanocomposites: synergistic collaboration on visible-light-driven activity in photodegradation of an organic pollutant. Appl Surf Sci..

[74] Yan T et al (2014) Facile fabrication of heterostructured g-C3N4/Bi2MoO6 microspheres with highly efficient activity under visible light irradiation. Dalt. Trans 10.1039/c4dt02127d 25428510

[75] Lv J (2015). Facile synthesis of Z-scheme graphitic-C 3 N 4 / Bi 2 MoO 6 nanocomposite for enhanced visible photocatalytic properties. Appl Surf Sci..

[76] Song L, Pang Y, Zheng Y, Ge L (2017). Hydrothermal synthesis of novel g-C3N4/BiOCl heterostructure nanodiscs for efficient visible light photodegradation of Rhodamine B. Appl Phys A Mater Sci Process..

[77] He R, Zhou J, Fu H, Zhang S, Jiang C (2018). Room-temperature in situ fabrication of Bi2O3/g-C3N4 direct Z-scheme photocatalyst with enhanced photocatalytic activity. Appl Surf Sci..

[78] Zhang J, Hu Y, Jiang X, Chen S, Meng S, Fu X (2014). Design of a direct Z-scheme photocatalyst: preparation and characterization of Bi2O3/g-C3N4 with high visible light activity. J Hazard Mater..

[79] Zhao J, Yan J, Jia H, Zhong S, Zhang X, Xu L (2016). BiVO4/g-C3N4composite visible-light photocatalyst for effective elimination of aqueous organic pollutants. J Mol Catal A Chem..

[80] Cui Y (2018). Construction of Bi2O3/g-C3N4composite photocatalyst and its enhanced visible light photocatalytic performance and mechanism. Sep Purif Technol.

[81] Liu S, Chen J, Xu D, Zhang X, Shen M (2018) Enhanced photocatalytic activity of direct Z-scheme Bi2O3/g-C3N4composites via facile one-step fabrication. J Mater Res

[82] Chen S, Hu Y, Jiang X, Meng S, Fu X (2015). Fabrication and characterization of novel Z-scheme photocatalyst WO3/g-C3N4with high efficient visible light photocatalytic activity. Mater Chem Phys..

[83] Gondal MA (2015). Preparation of WO3/g-C3N4 composites and their enhanced photodegradation of contaminants in aqueous solution under visible light irradiation. React Kinet Mech Catal..

[84] Huang L (2013). Visible-light-induced WO3/g-C3N4 composites with enhanced photocatalytic activity. Dalt Trans.

[85] Huang L (2013). Synthesis and characterization of g-C3N4/MoO3photocatalyst with improved visible-light photoactivity. Appl Surf Sci..

[86] Liu W, Zhou J, Hu Z, Zhou J, Cai W (2018). In situ facile fabrication of Z-scheme leaf-like β-Bi2O3/g-C3N4 nanosheets composites with enhanced visible light photoactivity. J Mater Sci Mater Electron..

[87] Long G (2018). Fabrication of mediator-free g-C3N4/Bi2WO6 Z-scheme with enhanced photocatalytic reduction dechlorination performance of 2,4-DCP. Appl Surf Sci.

[88] Meng S, Ning X, Zhang T, Chen SF, Fu X (2015). What is the transfer mechanism of photogenerated carriers for the nanocomposite photocatalyst Ag3PO4/g-C3N4, band-band transfer or a direct Z-scheme?. Phys Chem Chem Phys..

[89] Na Tian YZ, Huang H, He Y, Guo Y, Zhang T (2010). Mediator-free direct Z-scheme photocatalytic system: BiVO4/g-C3N4 organic-inorganic hybrid photocatalyst with highly efficient visible-light-induced photocatalytic activity. Optoelectron Adv Mater Rapid Commun..

[90] Xiao T, Tang Z, Yang Y, Tang L, Zhou Y, Zou Z (2018). In situ construction of hierarchical WO3/g-C3N4composite hollow microspheres as a Z-scheme photocatalyst for the degradation of antibiotics. Appl Catal B Environ..

[91] J. L. and W. X. Jiaguo Yu, Shuhan Wang, “Enhanced photocatalytic performance of direct Z-scheme g-C3N4/TiO2 photocatalyst for decomposition of formaldehyde in air,” Phys. Chem. Chem. Phys., vol. 13, no. 22, pp. 6397–6406, 2013. 10.1039/c3cp53131g 23999576

[92] Zhang J, Qian H, Liu W, Chen H, Qu Y, Lin Z (2018). The construction of the heterostructural Bi 2 O 3 /g-C 3 N 4 composites with an enhanced photocatalytic activity. Nanobr Reports Rev.

[93] Huang Z, Sun Q, Lv K, Zhang Z, Li M, Li B (2015). Effect of contact interface between TiO2 and g-C3N4 on the photoreactivity of g-C3N4/TiO2 photocatalyst: (001) vs (101) facets of TiO2. Appl Catal B Environ..

[94] Shi L, Liang L, Wang F, Liu M, Sun J (2015). Enhanced visible-light photocatalytic activity and stability over g-C3N4/Ag2CO3 composites. J Mater Sci..

[95] Geng X, Chen S, Lv X, Jiang W, Wang T (2018). Synthesis of g-C 3 N 4 / Bi 5 O 7 I microspheres with enhanced photocatalytic activity under visible light. Appl Surf Sci.

[96] Hong Y (2016). In-situ synthesis of direct solid-state Z-scheme V2O5/g-C3N4 heterojunctions with enhanced visible light efficiency in photocatalytic degradation of pollutants. Appl Catal B Environ..

[97] Li FT (2015). Enhanced visible-light photocatalytic activity of active Al2O3/g-C3N4 heterojunctions synthesized via surface hydroxyl modification. J. Hazard Mater..

[98] Li J (2016). Synthesis of MoS2/g-C3N4 nanosheets as 2D heterojunction photocatalysts with enhanced visible light activity. Appl Surf Sci..

[99] Duan Y (2018). Facile preparation of CuO/g-C3N4 with enhanced photocatalytic degradation of salicylic acid. Mater Res Bull..

[100] Zuo S (2018). “Molten-salt synthesis of g-C3N4-Cu2O heterojunctions with highly enhanced photocatalytic performance,” Colloids Surfaces A Physicochem. Eng Asp.

[101] Zhou X (2018). Heterojunction of g-C3N4/BiOI immobilized on flexible electrospun polyacrylonitrile nanofibers: facile preparation and enhanced visible photocatalytic activity for floating photocatalysis. ACS Sustain Chem Eng..

[102] W. Y. & M. L. Bin Ren, Tiecheng Wang, Guangzhou Qu, Fang Deng, Dongli Liang, “Insitu synthesis of g-C3N4/TiO2 heterojunction nanocomposites as a highly active photocatalyst for the degradation of Orange II under visible light irradiation,” Environ Sci Pollut Res*.*, vol. 25, pp. 19122–19133, 2018. 10.1007/s11356-018-2114-z 29725923

[103] Li J (2017). Insitu growing Bi2MoO6 on g-C3N4 nanosheets with enhanced photocatalytic hydrogen evolution and disinfection of bacteria under visible light irradiation. J Hazard Mater..

[104] Qiao Q, Yang K, Ma L, Huang W, Zhou B (2018). Facile insitu construction of mediator- free direct Z-scheme g-C3N4/CeO2 heterojunctions with highly efficient photocatalytic activity. J Phys D Appl Phys.

[105] Miao X (2017). g-C3N4/AgBr nanocomposite decorated with carbon dots as a highly efficient visible-light-driven photocatalyst. J Colloid Interface Sci..

[106] Han X (2018). WO3/g-C3N4two-dimensional composites for visible-light driven photocatalytic hydrogen production. Int J Hydrogen Energy.

[107] Katsumata H, Tachi Y, Suzuki T, Kaneco S (2014). Z-scheme photocatalytic hydrogen production over WO _3_ /g-C _3_ N _4_ composite photocatalysts. RSC Adv..

[108] Settanni G (2016). Z-scheme photocatalytic hydrogen production over WO3/g-C3N4 composite photocatalysts.

[109] Li R (2015). Achieving overall water splitting using titanium dioxide-based photocatalysts of different phases. Energy Environ Sci..

[110] Li W, Feng C, Dai S, Yue J, Hua F, Hou H (2015). Fabrication of sulfur-doped g-C3N4/Au/CdS Z-scheme photocatalyst to improve the photocatalytic performance under visible light. Appl Catal B Environ..

[111] Chen W (2015). A novel yet simple strategy to fabricate visible light responsive C,N-TiO2/g-C3N4heterostructures with significantly enhanced photocatalytic hydrogen generation. RSC Adv..

[112] Akple MS, Low J, Wageh S, Al-Ghamdi AA, Yu J, Zhang J (2015). Enhanced visible light photocatalytic H2-production of g-C3N4/WS2composite heterostructures. Appl Surf Sci..

[113] She X (2017). High efficiency photocatalytic water splitting using 2D Α-Fe2O3/g-C3N4 Z-scheme catalysts. Adv Energy Mater..

[114] Yang L (2017). Combining photocatalytic hydrogen generation and capsule storage in graphene based sandwich structures. Nat Commun.

[115] Wang X (2019). Isolating hydrogen from oxygen in photocatalytic water splitting with a carbon-quantum-dot/carbon-nitride hybrid. J Mater Chem A.

[116] Manahan SE (2000). “The endangered global atmosphere,” in Environmental Chemistry, Seventh Ed..

[117] Chakravarty S, Chikkatur A, de Coninck H, Pacala S, Socolow R, Tavoni M (2009). Sharing global CO2 emission reductions among one billion high emitters. Proceedings of the National Academy of Sciences.

[118] Rogelj J (2015). Energy system transformations for limiting end-of-century warming to below 1.5 °C. Nat Clim Chang..

[119] Duguma LA, Am P, Minang v N (2014). Climate change mitigation and adaptation in the land use sector : from complementarity to synergy. Environ Manage.

[120] Xu Y, Ramanathan V (2017). Well below 2 °C: mitigation strategies for avoiding dangerous to catastrophic climate changes. Proc Natl Acad Sci.

[121] Solano Rodriguez B, Drummond P, Ekins P (2017). Decarbonizing the EU energy system by 2050: an important role for BECCS. Clim Policy.

[122] Fuglestvedt J et al (2018) Implications of possible interpretations of’greenhouse gas balance’ in the Paris Agreement. Philos Trans R Soc A Math Phys Eng Sci 376(2119) 10.1098/rsta.2016.0445 PMC5897819 29610378

[123] Duscha V, Denishchenkova A, Wachsmuth J (2018). Achievability of the Paris Agreement targets in the EU: demand-side reduction potentials in a carbon budget perspective. Clim Policy.

[124] Yu W, Xu D, Peng T (2015). Enhanced photocatalytic activity of g-C3N4 for selective CO2reduction to CH3OH via facile coupling of ZnO: A direct Z-scheme mechanism. J Mater Chem A.

[125] He Y (2015). Z-scheme SnO2-x/g-C3N4composite as an efficient photocatalyst for dye degradation and photocatalytic CO2reduction. Sol Energy Mater Sol Cells.

[126] Zhou S (2014). Facile in situ synthesis of graphitic carbon nitride ( g-C 3 N 4 ) -N-TiO 2 heterojunction as an efficient photocatalyst for the selective photoreduction of CO 2 to CO. Applied Catal B Environ.

[127] Nie YC (2018). Photocatalytic degradation of organic pollutants coupled with simultaneous photocatalytic H2evolution over graphene quantum dots/Mn-N-TiO2/g-C3N4composite catalysts: performance and mechanism. Appl Catal B Environ.

[128] WK Darkwah**,** BB Adormaa, YAo Modification strategies for enhancing visible light responsive photocatalytic activity of BiPO4 nano base composite photocatalyst. Catalysis Science & Technology. (2019). Published by Royal Society of Chemistry 10.1039/C8CY02039F Accepted manuscript.

[129] BB Adormaa, WK Darkwah, YAo. Oxygen vacancy of TiO_2_ nano base composite photocatalyst in visible light responsive photocatalysis. RSC Advances. (2018).9. Published by Royal Society of Chemistry 10.1039/c8ra05117h PMC9086469 35548159

